# Mechanisms of ranolazine pretreatment in preventing ventricular tachyarrhythmias in diabetic *db/db* mice with acute regional ischemia–reperfusion injury

**DOI:** 10.1038/s41598-020-77014-0

**Published:** 2020-11-18

**Authors:** Chung-Chuan Chou, Hui-Ling Lee, Gwo-Jyh Chang, Hung-Ta Wo, Tzung-Hai Yen, Ming-Shien Wen, Yen Chu, Hao-Tien Liu, Po-Cheng Chang

**Affiliations:** 1 Division of Cardiology, Chang Gung Memorial Hospital, No. 5, Fu-Shing Street, Linkou, Gueishan, Taoyuan, 333 Taiwan; 2 Division of Nephrology, Department of Internal Medicine, Chang Gung Memorial Hospital, Linkou, Taiwan; 3 Department of Thoracic Surgery, Chang Gung Memorial Hospital, Linkou, Taiwan; 4 Department of Anesthesia, Chang Gung Memorial Hospital, Taipei, Taiwan; 5 Graduate Institute of Clinical Medicine, Taoyuan, Taiwan; 6 Chang Gung University College of Medicine, Taoyuan, Taiwan

**Keywords:** Experimental models of disease, Cardiovascular biology

## Abstract

Studies have demonstrated that diabetic (*db/db*) mice have increased susceptibility to myocardial ischemia–reperfusion (IR) injury and ventricular tachyarrhythmias (VA). We aimed to investigate the antiarrhythmic and molecular mechanisms of ranolazine in *db/db* mouse hearts with acute IR injury. Ranolazine was administered for 1 week before coronary artery ligation. Diabetic *db/db* and control *db/*+ mice were divided into ranolazine-given and -nongiven groups. IR model was created by 15-min left coronary artery ligation and 10-min reperfusion. In vivo electrophysiological studies showed that the severity of VA inducibility was higher in *db/db* mice than control (*db/* +) mice. Ranolazine suppressed the VA inducibility and severity. Optical mapping studies in Langendorff-perfused hearts showed that ranolazine significantly shortened action potential duration, Ca_i_ transient duration, Ca_i_ decay time, ameliorated conduction inhomogeneity, and suppressed arrhythmogenic alternans induction. Western blotting studies showed that the expression of pThr^17^-phospholamban, calsequestrin 2 and voltage-gated sodium channel in the IR zone was significantly downregulated in *db/db* mice, which was ameliorated with ranolazine pretreatment and might play a role in the anti-arrhythmic actions of ranolazine in *db/db* mouse hearts with IR injury.

## Introduction

Studies have demonstrated that diabetic (*db/db*) mice have increased susceptibility to myocardial ischemia–reperfusion (IR) injury^1,2^, a longer duration of IR-induced ventricular tachycardia (VT) and more degeneration of VT into ventricular fibrillation (VF)^3^, and a greater mortality after IR compared with control (*db/* +) mice^4^. However, the underlying electrophysiological and molecular mechanisms remain incompletely understood. Accumulation of intracellular Na^+^ occurs during IR^5^, and increased late sodium current (*I*_Na,L_) has been linked to elevated intracellular Na^+^ during IR. Upon reperfusion of ischemic myocardium, the sudden availability of oxygen in the ischemic myocardium increases the formation of reactive oxygen species which are known to increase *I*_Na,L_^6^, thereby worsening intracellular Na^+^ overload^7^. Subsequently, intracellular Ca^2+^ (Ca_i_) overload occurs via reverse-mode Na^+^/Ca^2+^ exchanger (NCX), leading to cell damage, apoptosis, and lethal cardiac arrhythmias. In diabetic mice, it has been reported that phosphoinositide 3-kinase signaling is reduced, resulting in a higher *I*_Na,L_ in cardiomyocytes from *db/db* mice than in wild-type cardiomyocytes^8^. A higher intrinsic *I*_Na,L_ density would play a role in the increased susceptibility to IR arrhythmias in *db/db* mice. Ranolazine, a clinically used nonspecific blocker of *I*_Na,L_^9^, was reported to reduce Ca^2+^ overload and oxidative stress, to improve mitochondrial integrity^10^, and to reduce ventricular tachyarrhythmia (VA) induced by IR injury^11^. In this study, we conducted simultaneous Ca_i_ and membrane voltage (V_m_) optical mapping to investigate the arrhythmogenicity of *db/db* mice with acute IR injury and the antiarrhythmic mechanisms of ranolazine in these hearts. We also performed immunoblot studies to investigate the molecular remodeling in correlation with the electrophysiological remodeling by acute regional IR injury with or without ranolazine treatment.

## Results

### Ranolazine suppressed in vivo VA inducibility and severity in mouse hearts with acute regional IR injury

**Figure 1:**
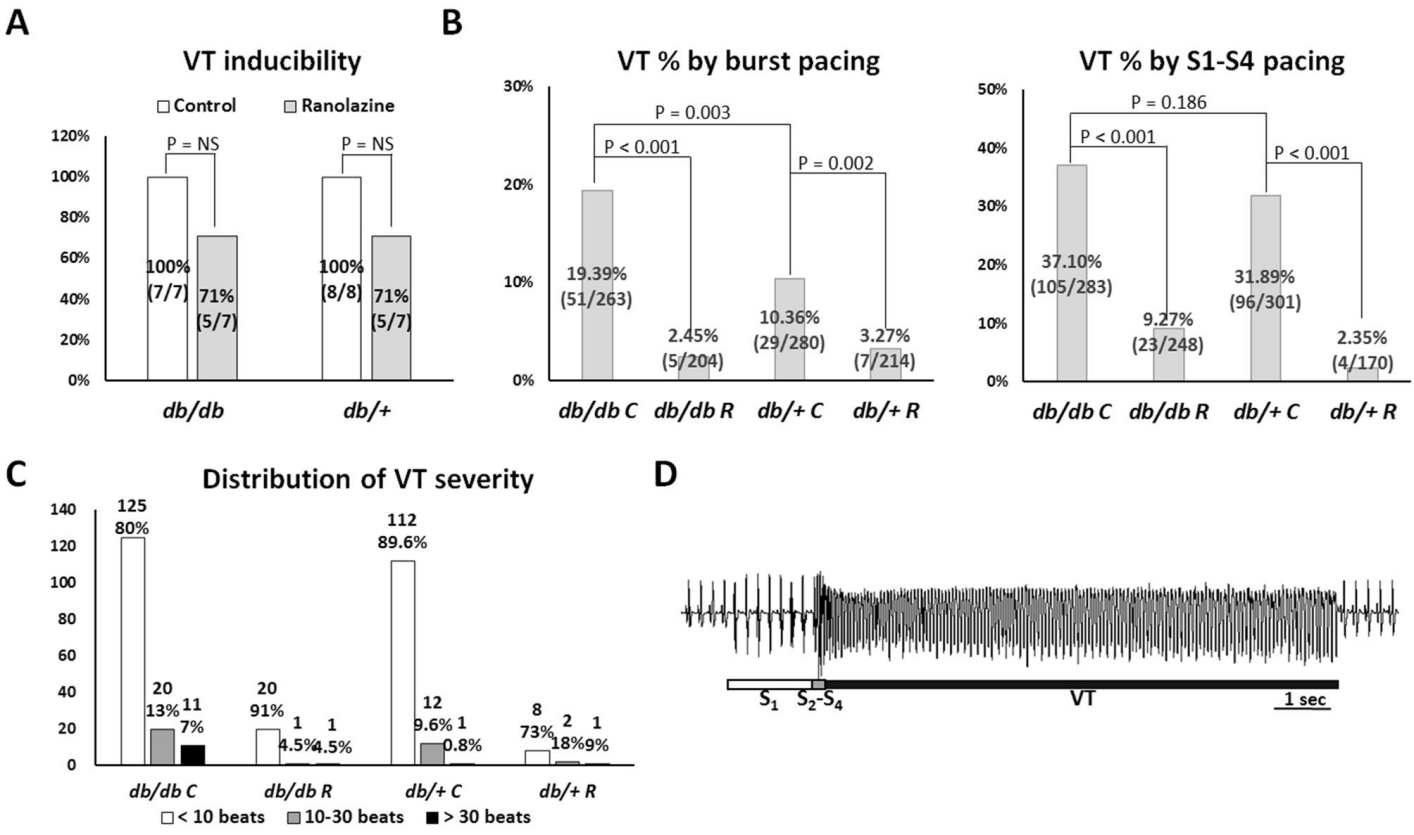
In vivo electrophysiological study. (**A**) Summary of in vivo ventricular tachycardia (VT) inducibility. (**B**) Percentage of VT episodes by pacing protocols. The number and percentage of pacing-induced VT were shown in the middle of each bar. Pretreatment of ranolazine significantly reduced the inducibility ratio of VT episodes by both burst pacing and extrastimulus pacing protocols in *db/db* and *db/*+ groups. (**C**) Distribution of the severity of VT, plotted as the number of beats of VT. The number and percentage of VT episodes were shown on the top of each bar. The *db/db* C group had higher percentage of long VT (> 30 beats) than other groups (*P* = 0.031). (**D**) A representative example of pseudo-electrocardiogram showing extrastimulus pacing-induced VT in a *db/db* C mouse heart.

In the in vivo electrophysiological studies, we acquired data from 7, 7, 8, and 7 mice in the *db/db* C, *db/db* R, *db/*+ C, and *db/*+ R groups, respectively. The effective refractory period was significantly longer in the *db/db* C mice than in the *db/db* R, *db/*+ C, and *db/*+ R groups (74 ± 17 vs. 62 ± 18, 58 ± 18, and 60 ± 11 ms, respectively; *P* = 0.034). Figure 1 summarizes the result of VT inducibility and severity. VT was inducible in 7 of 7, 5 of 7, 8 of 8 and 5 of 7 mice in the *db/db* C, *db/db* R, *db/*+ C, and *db/*+ R groups, respectively (*P* = NS, Fig. 1A). But the percentage of VT-induced episodes by burst pacing protocol was higher in the *db/db* C group compared to the *db/*+ C group (*P* = 0.003). Pretreatment of ranolazine significantly reduced the percentage of VT episodes by both burst pacing and extrastimulus pacing protocols in both *db/db* and *db/*+ groups (Fig. 1B). The distribution of VT episodes shown in Fig. 1C suggests that *db/db* C mice were significantly more vulnerable to long VT (> 30 beats), which was 11 episodes induced in 5 of 7 *db/db* C mice (the longest 180 beats), 1 episode induced in 1 of 7 *db/db* R mice (the longest 50 beats), 1 episode induced in 1 of 8 *db/*+ C mice (the longest 72 beats), and 1 episode induced in 1 of 7 *db/*+ R mice (the longest 66 beats) hearts (*P* = 0.031). A representative example of pacing-induced long VT in a *db/db* C mouse heart is shown in Fig. 1D.

### The electrophysiologic mechanisms of ranolazine in suppressing VA inducibility

#### *Ranolazine shortened and reduced dispersion of APD*_*80*_* and Ca*_*i*_*TD*_*80*_

**Figure 2:**
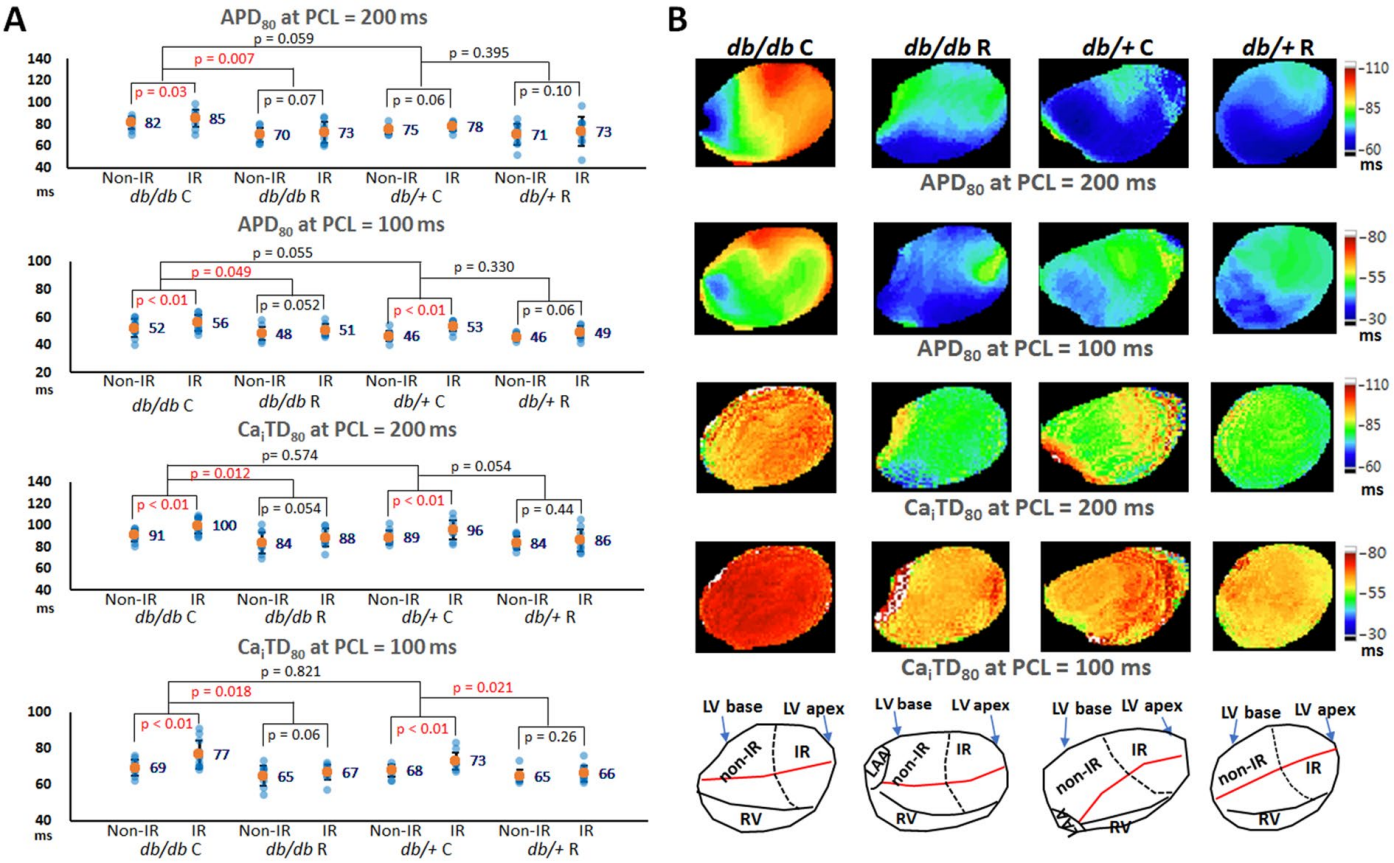
Effects of ranolazine therapy on APD_80_ and Ca_i_TD_80_. (**A**) Scattered graphs of APD_80_ and Ca_i_TD_80_ at pacing cycle length (PCL) of 200 ms and 100 ms. Orange dots and numbers indicate the mean values. Ranolazine significantly shortened APD_80_ and Ca_i_TD_80_ in *db/db* mice. (**B**) Representative APD_80_ and Ca_i_TD_80_ maps at pacing cycle length (PCL) = 200 and 100 ms.

In the optical mapping studies, we acquired data from 11, 10, 11, and 10 mice in the *db/db* C, *db/db* R, *db/*+ C, and *db/*+ R groups, respectively. Figure 2A summarizes the results. The *db/db* C group tended to have a longer APD_80_ than the *db/*+ C group. APD_80_ in the *db/db* C group was significantly longer than that in the *db/db* R group, but there was no significant difference between the *db/*+ C and *db/*+ R groups. In addition, APD_80_ in the IR zone was significantly longer than that in the non-IR zone in the ranolazine non-given groups, but not in the ranolazine given groups (Table 1). The APD_80_ dispersion was significantly different among the four groups and the *db/db* C group had the largest APD_80_ dispersion: at PCL = 200 ms: 27 ± 8, 22 ± 7, 17 ± 5, 16 ± 3 ms in *db/db* C, *db/*+ C, *db/db* R, *db/*+ R groups, respectively (*P* = 0.002); at PCL = 100 ms: 17 ± 5, 17 ± 3, 13 ± 3, 13 ± 4 ms in *db/db* C, *db/*+ C, *db/db* R, *db/*+ R groups, respectively (*P* = 0.041). Similarly, Ca_i_TD_80_ in the *db/db* C group was significantly longer than that in the *db/db* R group, and Ca_i_TD_80_ in the IR zone was significantly longer than that in the non-IR zone in the ranolazine non-given groups, but not in the ranolazine given groups (Table 1). The difference of Ca_i_TD_80_ dispersion was insignificant at PCL = 200 ms: 23 ± 7, 19 ± 7, 17 ± 8, and 16 ± 6 ms (*P* = 0.217); but was significant at PCL = 100 ms: 18 ± 7, 18 ± 7, 10 ± 5, and 11 ± 5 ms (*P* = 0.028) in the *db/db* C, *db/*+ C, *db/db* R, and *db/*+ R groups, respectively. These findings indicated that ranolazine shortened APD_80_ and Ca_i_TD_80_, reduced the differences of APD_80_ and Ca_i_TD_80_ between non-IR and IR zones, and attenuated the APD_80_ and Ca_i_TD_80_ heterogeneity in both *db/db* and *db/*+ mouse hearts. Figure 2B shows representative examples of APD_80_ and Ca_i_TD_80_ maps of the four groups. The *db/db* C mouse heart had the longest APD_80_ and Ca_i_TD_80_, which were longer in the IR zone than in the non-IR zone.

**Table 1:** Electrophysiological effects of ranolazine in isolated Langendorff-perfused mouse hearts after IR injury.

	APD_80_ (ms)		Ca_i_TD_80_ (ms)		CV (cm/s)							
	200 ms	100 ms	200 ms	100 ms	200 ms	150 ms	120 ms	100 ms	90 ms	80 ms	70 ms	60 ms
***db/db*** **C (N = 11)**												
Non-IR	82 ± 9*	52 ± 7*	91 ± 7*	69 ± 5*	78 ± 13*	76 ± 13*	74 ± 14*	70 ± 13*	64 ± 12*	57 ± 13*	51 ± 12*	45 ± 12*
IR	86 ± 9*	56 ± 6*	100 ± 9*	77 ± 8*	68 ± 18*	61 ± 16*	57 ± 15*	54 ± 14*	50 ± 12*	47 ± 11*	41 ± 10*	37 ± 10*
***db/db*** **R (N = 10)**												
Non-IR	70 ± 7	48 ± 6	84 ± 11	65 ± 6	88 ± 5	84 ± 8	80 ± 9	75 ± 8	74 ± 11	72 ± 12	64 ± 12	62 ± 10
IR	73 ± 10	51 ± 6	89 ± 9	67 ± 5	82 ± 9	78 ± 8	76 ± 7	70 ± 6	67 ± 7	61 ± 6	58 ± 5	53 ± 6
***db/*+ C (N = 11)**												
Non-IR	75 ± 4	46 ± 4*	89 ± 7*	68 ± 3*	83 ± 15	78 ± 13*	75 ± 10*	70 ± 12*	67 ± 13*	60 ± 12*	57 ± 13*	52 ± 11*
IR	78 ± 4	54 ± 4*	96 ± 10*	73 ± 6*	69 ± 12	64 ± 13*	59 ± 11*	55 ± 9*	51 ± 9*	46 ± 8*	43 ± 7*	38 ± 7*
***db/*+ R (N = 10)**												
Non-IR	71 ± 10	46 ± 2	84 ± 6	65 ± 4	90 ± 25	89 ± 25	85 ± 27	83 ± 28	80 ± 25	75 ± 23	67 ± 19	61 ± 17
IR	73 ± 14	49 ± 5	86 ± 10	66 ± 5	86 ± 22	82 ± 21	80 ± 22	75 ± 24	72 ± 21	68 ± 22	64 ± 21	56 ± 16

Values are mean ± SD. APD_80_, action potential duration at 80% repolarization; Ca_i_TD_80_, effective refractory period; CV, conduction velocity; IR, ischemia–reperfusion. * indicates *P* < 0.05 for non-IR vs. IR.

#### *Ranolazine fastened Ca*_*i*_* decay*

**Figure 3:**
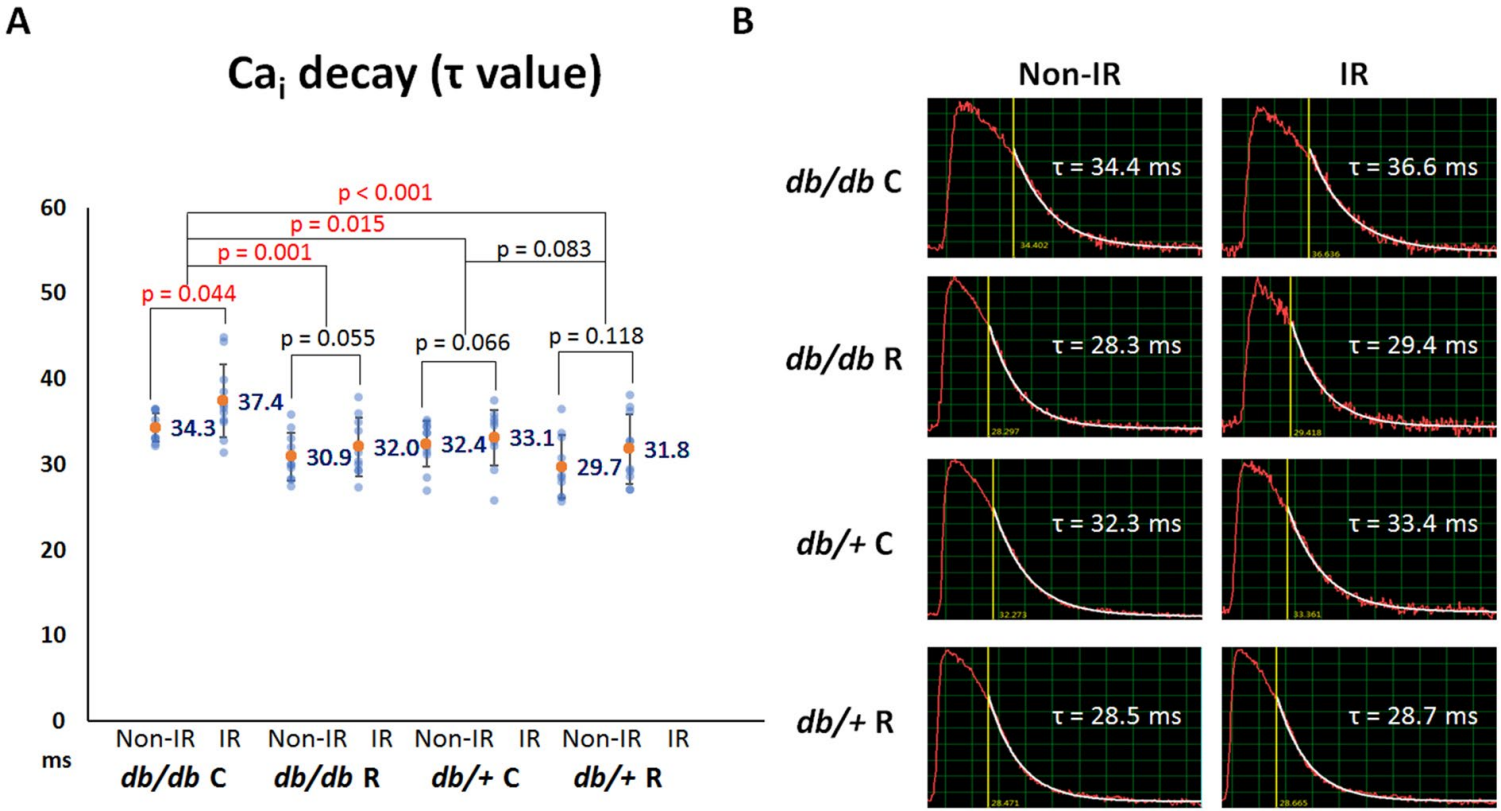
Effects of ranolazine therapy on intracellular Ca^2+^ (Ca_i_) decay. (**A**) Scattered graphs of Ca_i_ decay tau values among the four groups and between the ischemia–reperfusion (IR) and non-IR zones. Orange dots and numbers indicate the mean values. Ranolazine therapy shortened the tau value in *db/db* mice and ameliorated the differences of tau values between the non-IR and IR zones in *db/db* mice. (**B**) Representative examples of Ca_i_ decay in the non-IR and IR zones among the four groups.

Dysregulation of Ca_i_ homeostasis plays a role in the development of IR-induced VA. Figure 3A summarizes the results of Ca_i_ decay tau value among the four groups. Ca_i_ decay time was the longest in the *db/db* C group among the four groups (*P* = 0.013). The post hoc analysis shows that ranolazine shortened the tau value significantly in *db/db* mouse hearts (from 35.8 ± 3.8 ms to 31.5 ± 3.4 ms; *P* = 0.001) but insignificant in *db/*+ mouse hearts (from 32.8 ± 3.5 ms to 30.8 ± 3.9 ms; *P* = 0.083). Furthermore, Ca_i_ decay time was longer in the IR zone than in the non-IR zone. Ranolazine ameliorated the differences in the tau values between the non-IR and IR zones in *db/db* mice. As shown in Fig. 3A, the *P* values were increased from 0.044 to 0.055 (*db/db* C vs. *db/db* R). But the differences in the tau values between the non-IR and IR zones were insignificant in the *db/*+ C and *db/*+ R groups (*P* = 0.066 and 0.118, respectively). A representative example of Ca_i_ decay at the non-IR and IR zones in the four groups is shown in Fig. 3B.

#### Ranolazine ameliorated conduction inhomogeneity

**Figure 4:**
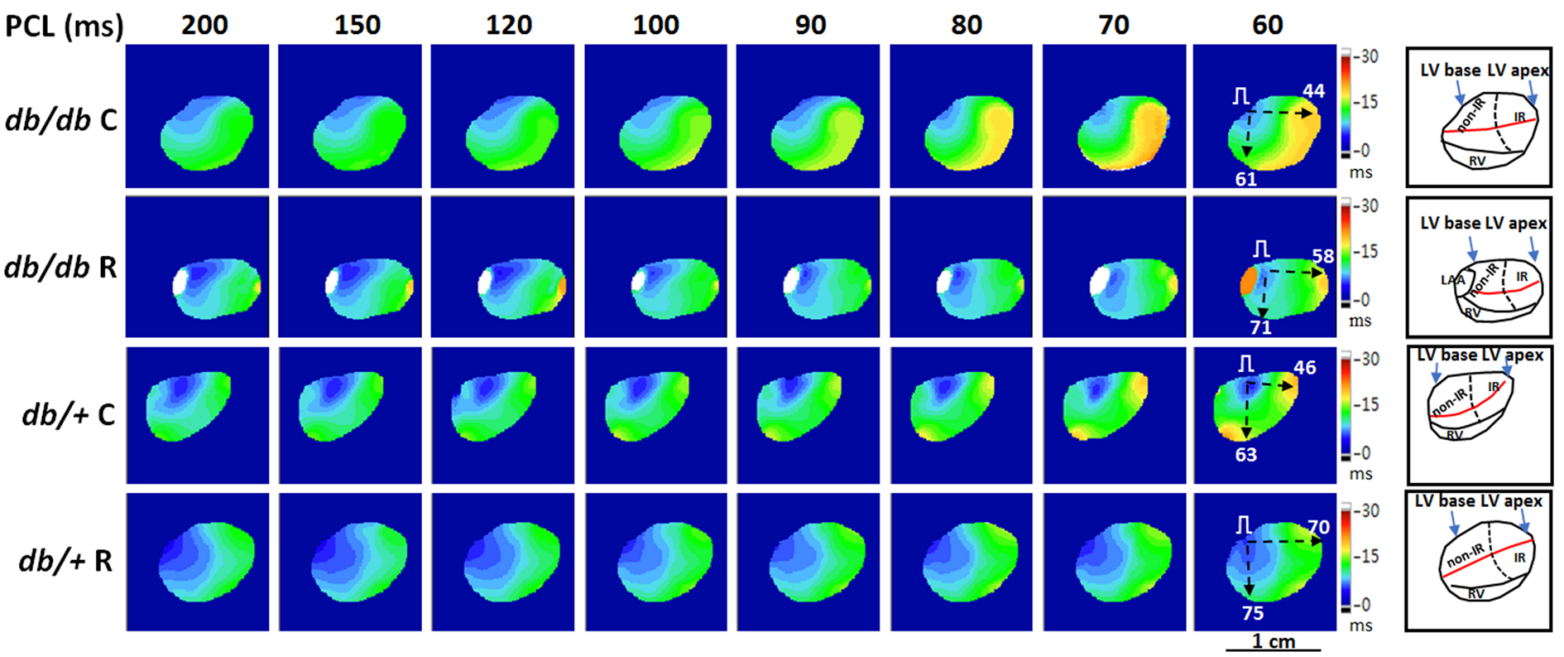
Representative examples of isochrone maps at a pacing cycle length (PCL) from 200 to 60 ms in four groups. Black dashed arrows indicate the directions of CV_non-IR_ (conduction velocity (CV) along the atrioventricular ring) and CV_IR_ (CV pointing to the apical ischemia–reperfusion zone) measurements; numbers in right subpanels are CV (cm/s). Right subpanels show the anatomical structure and orientation of optical maps. Ranolazine therapy ameliorated CV_IR_ slowing to ameliorate conduction inhomogeneity (see Table 1). Red line, left coronary artery; dashed line, margin of IR zone; LAA, left atrial appendage; LV, left ventricle; RV, right ventricle.

Table 1 summarizes the effects of 1-week ranolazine pretreatment on CV in mouse hearts with acute regional IR injury. The difference between CV_IR_ and CV_non-IR_ was significant in the *db/db* C and *db/*+ C groups, but was insignificant in the *db/db* R and *db/*+ R groups, suggesting that ranolazine ameliorated CV_IR_ slowing to ameliorate conduction inhomogeneity. Figure 4 shows an example of isochrone maps in the four groups. At PCL = 60 ms, the CV_IR_ was slower in the *db/db* C (44 cm/s) and *db/*+ C (46 cm/s) mice than in the *db/db* R (58 cm/s) and *db/*+ R (70 cm/s) mice; and the difference between CV_IR_ and CV_non-IR_ was also greater in the *db/db* C (17 cm/s) and *db/*+ C (17 cm/s) mice than in the *db/db* R (13 cm/s) and *db/*+ R (5 cm/s) mice.

#### Ranolazine suppressed induction of spatially concordant and discordant alternans

Alternans represents a phenomenon of electrophysiological instability which may lead to conduction block, re-entry, and tachyarrhythmias. Although spatially concordant alternans (SCA) could be provoked in all hearts in the four groups, the longest PCL required to provoke SCA was significantly shorter in the ranolazine groups (107 ± 15, 86 ± 5 ms in the *db/db* C, *db/db* R groups, respectively; 102 ± 15, 79 ± 17 ms in the *db/*+ C, *db/*+ R groups, respectively; *P* < 0.001). Similarly, spatially discordant alternans (SDA) could be provoked in all hearts, and the longest PCL required to provoke SDA was significantly shorter in the ranolazine groups (90 ± 12, 69 ± 11 ms in the *db/db* C, *db/db* R groups, respectively; 80 ± 14, 67 ± 9 ms in the *db/*+ C, *db/*+ R groups, respectively; *P* < 0.001). A representative example was shown in Supplementary Fig. S1.

#### Ranolazine suppressed VA inducibility

**Figure 5:**
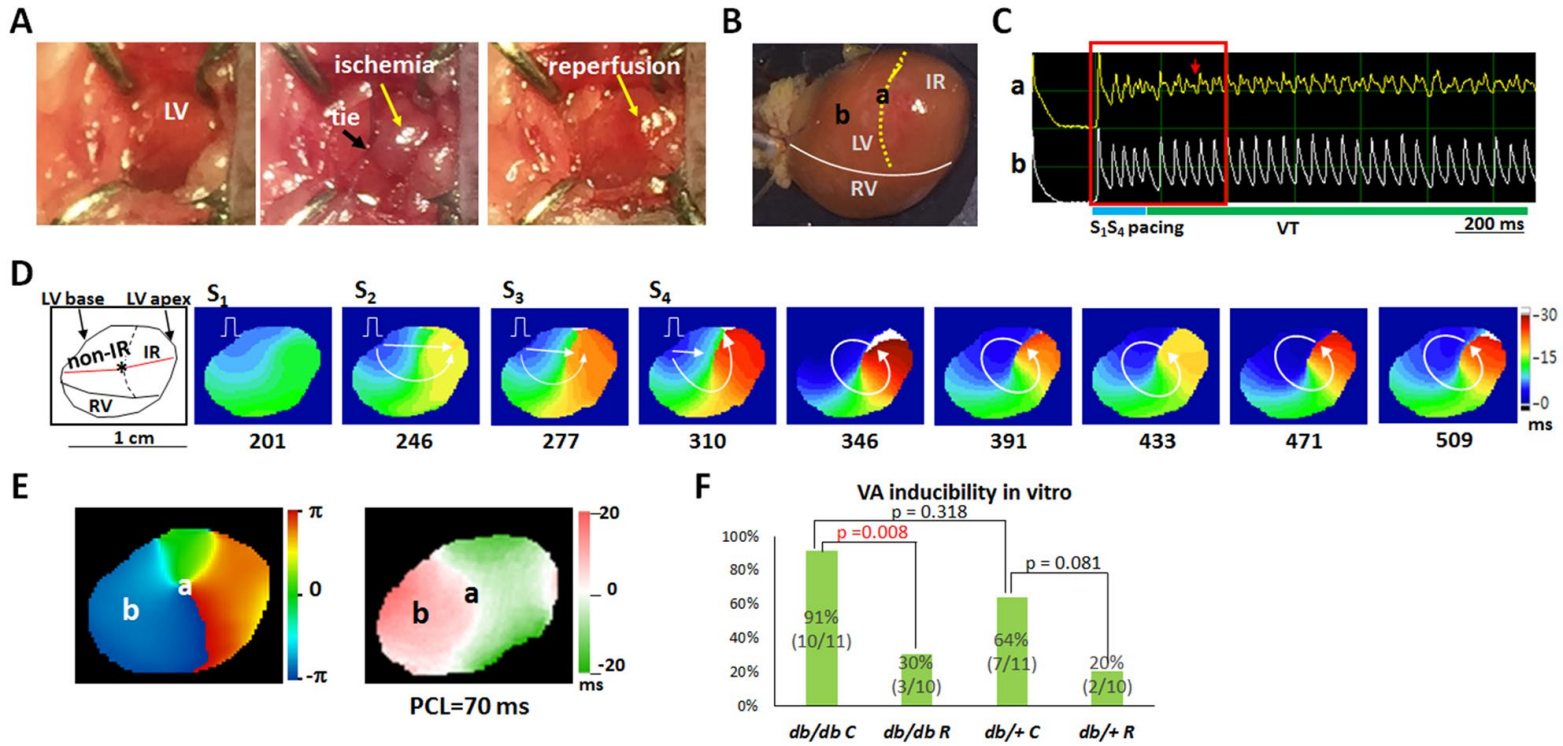
Mechanisms of ventricular tachycardia (VT) induction in a *db/db* C mouse heart with ischemia–reperfusion (IR) injury. (**A**) IR creation. Ischemia zone distal to the tie is shown in gray, and recovered after removal of the ligature in red. (**B**) Mapping field. Yellow dotted line indicates the margin of the IR zone. LV, left ventricle; RV, right ventricle. (**C**) Membrane voltage (V_m_) traces showing the initiation of VT by S_1_-S_4_ pacing. Red arrow indicates fragmented V_m_ transient during rotor anchoring at site “a”. (**D**) Isochrone maps corresponding to the period marked by a red square in C. The number below each frame is the time (ms) with the onset of data acquisition as time zero. White arrows indicate the directions of wavefront propagation. Left subpanel shows the anatomical structure of the mapping filed. Red line, left coronary artery; dashed line, margin of IR zone. (**E**) Phase singularity (*left*) and V_m_ alternans (*right*) maps. A phase singularity (site “a”) was formed on a nodal line during VT. (F) Summary of the VA inducibility result.

The *db/db* C group had the highest VA inducibility among the four groups: VA was induced in 10 of 11 (91%, *db/db* C), 3 of 10 (30%, *db/db* R), 7 of 11 (64%, *db/*+ C) and 2 of 10 (20%, *db/*+ R) hearts (*P* = 0.004). The VA inducibility was significantly different between the *db/db* C and *db/db* R groups (*P* = 0.008) but insignificantly different between the *db/*+ C and *db/*+ R groups (*P* = 0.081). Figure 5 illustrates VT induction in a *db/db* C mouse heart. Figure 5A,B show images of IR creation and the mapping field, respectively. Figure 5C shows the V_m_ recordings at sites “a” (rotor anchoring site on a nodal line, Fig. 5E) and “b” (left ventricular base) during VT induction. Extrastimulus pacing led to dispersion of refractoriness and unidirectional conduction block (frame 310; Fig. 5D), and reentrant wavefronts were initiated after pacing (frames 346–509). During the initiation of VT, the core of reentrant wavefronts anchored at site “a,” where fragmented V_m_ transient is shown (Fig. 5C). Post hoc analysis revealed that ranolazine effectively suppressed the VA inducibility in both *db/db* and *db/*+ mouse hearts with acute regional IR injury (Fig. 5F).

### Alteration of protein expression after acute regional IR injury

**Figure 6:**
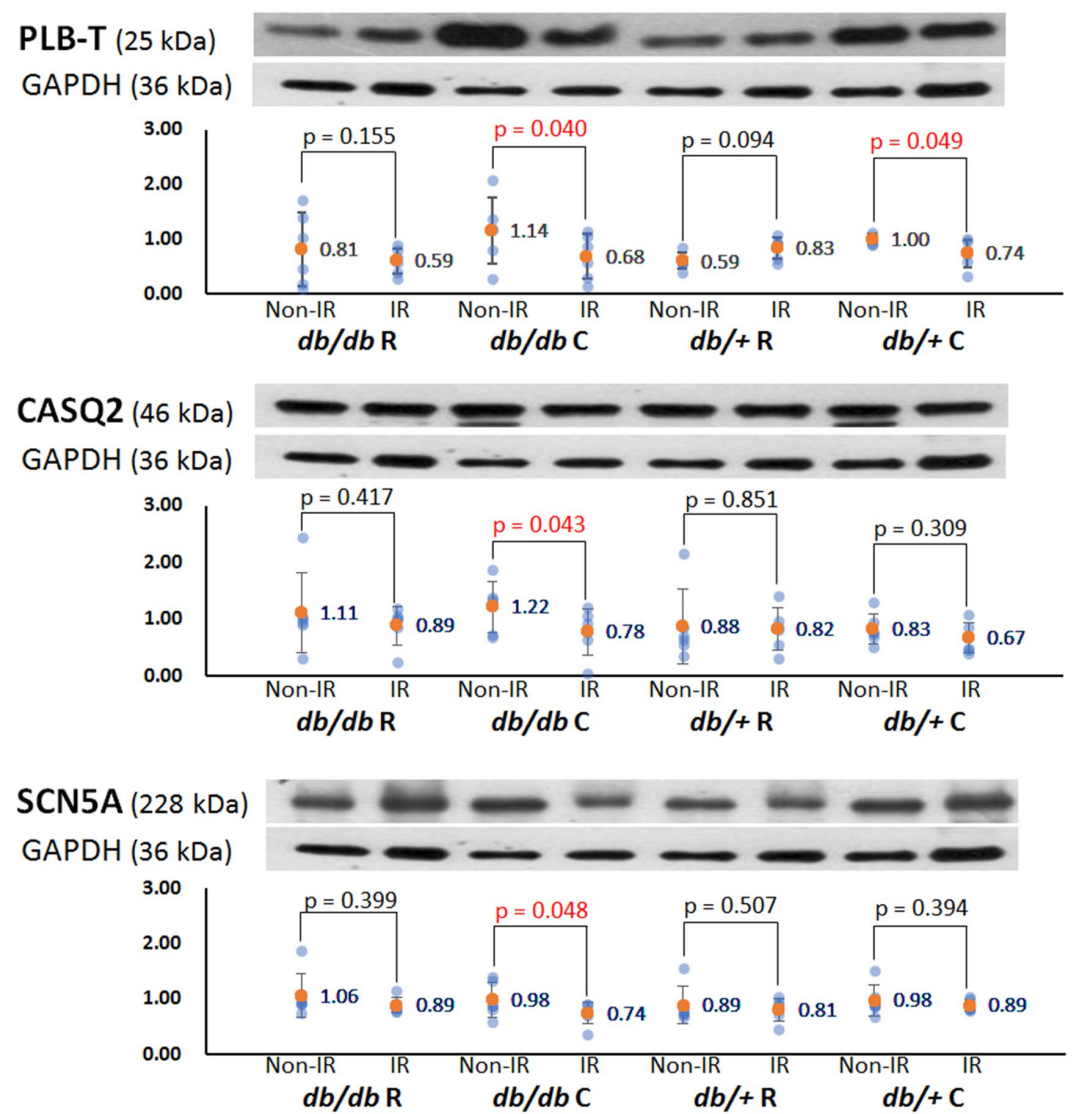
Representative examples of the Western blots result of pThr^17^-phospholamban (PLB-T), calsequestrin 2 (CASQ2) and voltage-gated sodium channel (SCN5A). Scattered graphs represent densitometric values normalized to the corresponding GAPDH. Orange dots and numbers indicated the mean values.

To elucidate the roles of Ca^2+^-handling proteins, Na^+^ channel, and Cx43 in the antiarrhythmic mechanisms of ranolazine, we measured and compared the levels of the associated proteins between the IR and non-IR zones. The results are presented in Fig. 6 and supplementary Fig. S2, and all analyzed proteins with fuller-length blots are shown in Supplementary Fig. S3. In *db/db* C hearts, the expression levels of pThr^17^-phospholamban, calsequestrin 2, and voltage-gated sodium channel (SCN5A) in the IR zone were significantly lower than those in the non-IR zone. Ranolazine pretreatment attenuated the downregulation of these proteins in the IR zone by acute IR injury. In *db/*+ C hearts, the expression level of pThr^17^-phospholamban was significantly lower than that in the non-IR zone, which was attenuated by ranolazine.

### Ranolazine effects on ***I***_Na,L_ in cardiomyocytes from ***db/db*** and ***db/***+ mice with acute regional IR injury

**Figure 7:**
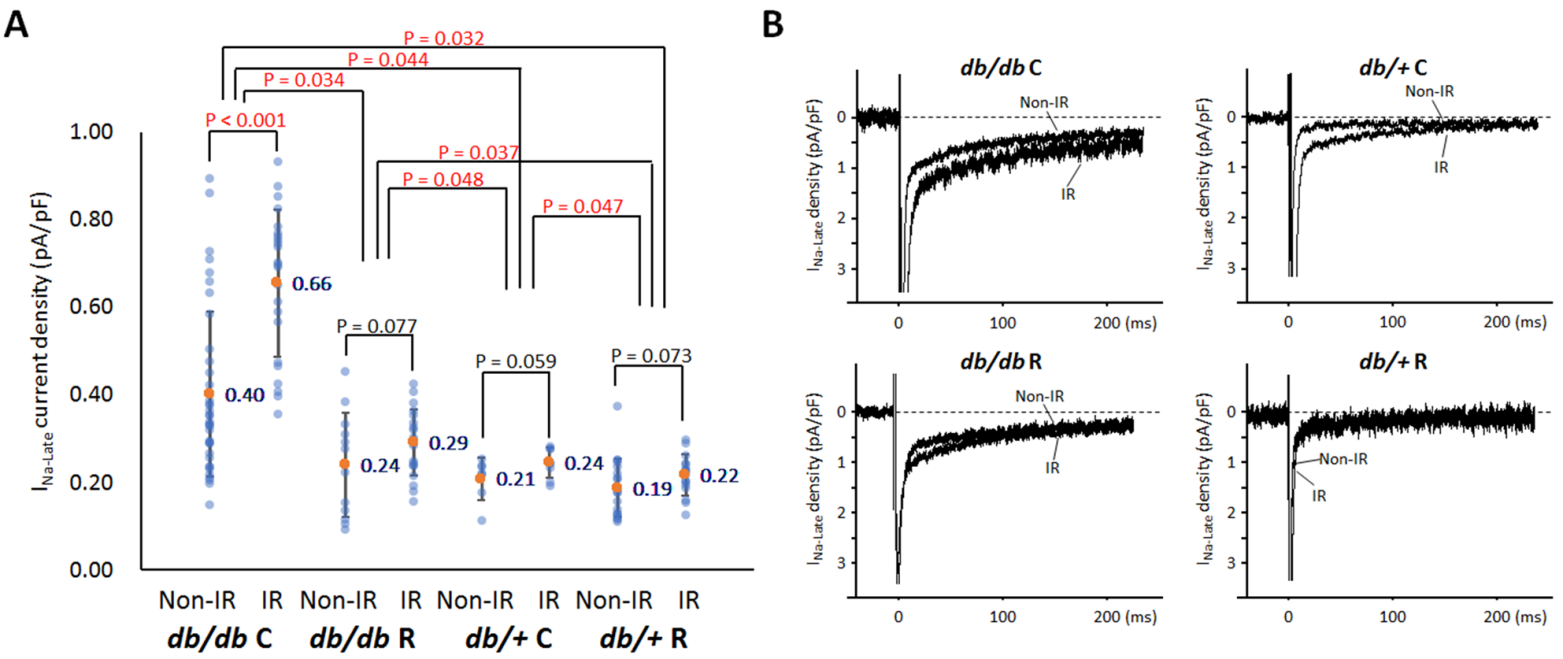
Whole-cell late Na^+^ currents (*I*_Na,L_) recording. (**A**) Scattered graphs of mean *I*_Na,L_. The graph shows the comparisons of the current density among four groups and between the ischemia–reperfusion (IR) and non-IR zones. Orange dots and numbers indicated the mean values. Student’s *t*-test for comparisons between the IR and non-IR zones and one-way repeated measures analysis of variance with post hoc least significant difference analysis for comparisons among the four groups. The *db/db* cardiomyocytes expressed a greater *I*_Na,L_ density than the *db/*+ cells. Ranolazine therapy significantly decreased the density of *I*_Na,L_ in both *db/db* mice and *db/*+ mice. (**B**) Representative *I*_Na,L_ traces of the cardiomyocytes among the four groups.

The *db/db* cardiomyocytes expressed a greater *I*_Na,L_ density (0.420 ± 0.214 pA/pF, n = 93) than the *db/*+ cells (0.209 ± 0.056 pA/pF, n = 51, *P* < 0.001). As shown in Fig. 7A, ranolazine therapy significantly decreased the density of *I*_Na,L_ in both *db/db* mice (0.497 ± 0.219 pA/pF, n = 61 in *db/db* C vs. 0.272 ± 0.096 pA/pF, n = 32 in *db/db* R, *P* = 0.034) and *db/*+ mice (0.229 ± 0.044 pA/pF, n = 15 in *db/*+ C vs. 0.201 ± 0.059 pA/pF, n = 36 in *db/*+ R, *P* = 0.047), but the density of *I*_Na,L_ in *db/db* R group was still higher than those in *db/*+ C (*P* = 0.048) and db/+ R (*P* = 0.037) groups. There was significant difference of *I*_Na,L_ density between IR and non-IR cardiomyocytes in the *db/db* C group (0.655 ± 0.168 pA/pF, n = 23 vs 0.402 ± 0.189 pA/pF, n = 38, *P* < 0.001), but not in the *db/db* R group (0.291 ± 0.076 pA/pF, n = 20 vs 0.240 ± 0.120 pA/pF, n = 12, *P* = 0.077). Representative I_*Na,L*_ recordings were shown in Fig. 7B.

## Discussion

Previously Ogawa et al. showed that acute ranolazine perfusion facilitated the termination of ischemic VT/VF by the suppression of *I*_Na,L_-dependent focal arrhythmogenic activity in isolated rabbit hearts^12^. Dhalla et al. reported that ranolazine markedly reduced IR-induced VAs possibly via its *I*_Na,L_ inhibitor property to reduce afterdepolarizations^11^. In this study our optical mapping results showed that 1-week ranolazine pretreatment significantly reduced VA inducibility via amelioration of IR injury-induced conduction inhomogeneity and impaired Ca_i_ decay, reduction of APD_80_ and Ca_i_TD_80_ prolongation and dispersion, and suppression of arrhythmogenic alternans induction. The in vivo electrophysiological studies show that the *db/db* C group was more vulnerable to long VT compared with the other three groups, suggesting that ranolazine pretreatment is effective in protecting *db/db* mice from IR-induced life-threatening VA. Western blotting showed that protein expression of pThr^17^-phospholamban, calsequestrin 2, and SCN5A was significantly decreased in the IR zone in diabetic mouse hearts, and ranolazine ameliorated the downregulation of these proteins. These molecular mechanisms may play a role in the antiarrhythmic actions of ranolazine in diabetic mouse hearts with regional IR injury. The whole-cell patch clamp study further confirmed that *db/db* cardiomyocytes expressed a greater *I*_Na,L_ density, which was significantly higher in the IR zone than the non-IR zone. Ranolazine significantly decreased the density of *I*_Na,L_ and reduced the difference of *I*_Na,L_ density between the IR and non-IR zones in *db/db* mouse hearts.

### Ranolazine administration improves Ca_i_ dynamics in the IR zone

During IR injury, Ca_i_ overload can result from the impaired ability of sarcoendoplasmic reticular Ca^2+^-ATPase (SERCA2a) to sequester cytosolic Ca^2+^ in stunned myocardium^13^, and from the enhanced *I*_Na,L_ to increase Na^+^ influx, and via reverse-mode NCX, to increase Ca_i_^9^. *I*_Na,L_ enhancement may also result in calcium/calmodulin protein kinase II (CaMKII) activation, which may induce proarrhythmic sarcoplasmic reticulum (SR) Ca^2+^ leak^14^. It has been reported that mitochondrial Ca^2+^ uptake, binding of the L-type Ca^2+^ channel to the sarcolemma, and Ca^2+^ intake by SR are decreased in diabetic hearts^15^. In conjunction with an intrinsic higher *I*_Na,L_, cellular dysregulation of Ca^2+^ homeostasis would be more pronounced in post-IR myocardial dysfunction and arrhythmogenicity in *db/db* mouse hearts.

Blockade of *I*_Na,L_ may reverse the impaired Ca^2+^ cycling in conditions of increased *I*_Na,L_. It has been shown that enhancement of *I*_Na,L_ increases the vulnerability to Ca_i_ alternans during rapid pacing^16^. Fukaya et al. reported that ranolazine reduces diastolic Ca_i_ and mitigates cardiac alternans, representing a mechanism underlying the antiarrhythmic benefit of *I*_Na,L_ blockade in heart failure^17^. Consistently, our data showed that ranolazine suppressed the induction of SCA and SDA in mouse hearts with IR injury.

In addition to *I*_Na,L_ blockade to ameliorate Ca_i_ overload, the presented data reveal some possible molecular mechanisms underlying the antiarrhythmic effects of ranolazine in the IR zone of diabetic hearts. Phospholamban is a key phosphorylation-dependent modulator of SERCA2a activity, and phospholamban dephosphorylation has been reported to account for myocardial stunning^18^. Our data show that ranolazine attenuated the downregulation of pThr^17^-phospholamban in the IR zone, which played a role in accelerating Ca_i_ decay and shortening Ca_i_TD_80_. The Ca_i_ alternans suppression and Ca_i_TD_80_ shortening were reported to reduce the susceptibility to subsequent refibrillation in a long-standing VF rabbit model^19^. Additionally, calsequestrin 2 is the main Ca^2+^-binding protein of the SR, serving as an important regulator of Ca^2+^ to protect the heart against premature Ca^2+^ release and triggered arrhythmias^20^. Downregulation of calsequestrin 2 increases SR Ca^2+^ leak and arrhythmia susceptibility under stress^21^. Our data show decreased expression of calsequestrin 2 in the IR zone of diabetic hearts, which may partly account for the increased VA inducibility in the *db/db* C group. Parikh et al. reported that ranolazine stabilizes cardiac ryanodine receptors to inhibit Ca_i_ oscillations and early afterdepolarizations^22^. The amelioration of calsequestrin 2 downregulation in the IR zone by ranolazine may contribute to the reduced VA inducibility in ranolazine-pretreated *db/db* mice.

It was reported that cardiac IR injury is accompanied by a marked reduction in SR Ca^2+^-pump ATPase, Ca^2+^-uptake and Ca^2+^-release activities, and the mRNA levels for SR Ca^2+^-handling proteins such as SERCA2a, ryanodine receptor, calsequestrin and phospholamban were decreased in the ischemia-reperfused heart as compared with the non-ischemic control^23^. Our data also shows that protein expression of pThr17-phospholamban, calsequestrin 2, and SCN5A was significantly decreased in the IR zone in diabetic mouse hearts, and ranolazine ameliorated the downregulation of these proteins. We do not know the exact mechanisms. Upon reperfusion of ischemic myocardium, the sudden availability of oxygen in the ischemic myocardium increases the formation of reactive oxygen species and intracellular Ca^2+^ overload, which cause cell damage and apoptosis. Because ranolazine was reported to reduce oxidative stress and Ca^2+^ overload, and to improve mitochondrial integrity during IR^10^, these actions may underlie the mechanism of ameliorating IR injury, including the downregulation of pThr17-phospholamban, calsequestrin 2, and SCN5A by ranolazine pretreatment.

### Ranolazine administration ameliorates conduction inhomogeneity in regional IR injury

Studies have shown reduced cardiac conduction reserve in diabetic animal models^24–26^. Therefore, propagation of activity through the myocardium in diabetic hearts is more sensitive to conditions influencing cellular excitability or intercellular electrical coupling. For example, more pronounced activation of Ca^2+^-independent phospholipase A_2_ in response to acute ischemia was reported to contribute to arrhythmogenic conduction slowing in the diabetic rat heart^27^. In the regional IR model, the elevated Ca_i_ in the IR myocardium may prolong refractoriness by stimulating NCX current and thereby prolong APD^28^, which interferes with wavefront propagation. The effect of ranolazine on APD depends on the relative contributions of *I*_Na,L_ and rapidly activating delayed rectifier potassium current to repolarization^9^. Ranolazine abbreviates APD and thereby refractoriness in conditions when *I*_Na,L_ is enhanced. Our data show that ranolazine shortened APD_80_, especially in the IR zone, which may conjoin with the attenuated downregulation of SCN5A in the IR zone to improve CV_IR_ in *db/db* mouse hearts. In addition, ranolazine, by shifting myocardial utilization of fatty acid to glucose during reperfusion, reduces deleterious lipid metabolites^29^. These lipid metabolites have been shown to cause uncoupling of gap junctions^30^. It is possible that ranolazine improves CV_IR_ via its beneficial effects on myocardial metabolism.

## Methods

This study protocol was approved by the Institutional Animal Care and Use Committee of Chang Gung Memorial Hospital (approval no. 2015092401) and conformed to the current NIH guidelines for the care and use of laboratory animals. The C57BL/KsJ strain was obtained from Jackson Laboratories (Bar Harbor, ME, USA) and grew in Taiwan. The mice were divided into four groups: diabetic mice not given ranolazine (*db/db* C, n = 22, 12 female, age 23.7 ± 3.6 weeks, body weight 55.0 ± 7.8 g), diabetic mice given ranolazine (db/db R, n = 21, 11 female, age 23.7 ± 5.5 weeks, body weight 59.6 ± 12.0 g), control mice not given ranolazine (*db/*+ C, n = 23, 11 female, age 23.2 ± 3.5 weeks, body weight 30.1 ± 3.9 g), and control mice given ranolazine (*db/*+ R, n = 21, 10 female, age 24.7 ± 2.9 weeks, body weight 31.9 ± 4.2 g). Ranolazine (R6152; Sigma-Aldrich, Munich, Germany) was administered orally at 305 mg/kg/d (dose comparable with that used clinically in humans of 750 mg twice daily)^31^ for 7 days.

### *In-vivo* IR model creation and electrophysiological studies

Mice were anesthetized with intra-peritoneal injection of xylazine (10 mg/kg) and zoletil (25 mg/kg). After mice appeared fully unconscious, endotracheal intubation was performed for gas general anesthesia with isoflurane (1%). Regional myocardial ischemia was induced by left coronary artery ligation at the midway between the atrio-ventricular junction and the apex. Ischemia was confirmed by the appearance of hypokinesis and pallor distal to the occlusion. After 15 min of ischemia, the ligature was removed, and reperfusion was visually confirmed (Fig. 5A).

In vivo electrophysiological study was performed after reperfusion for 10 min^24^. We first measured effective refractory period by giving a premature stimulus after 8 beats of S_1_S_1_ pacing at a pacing cycle length (PCL) of 200 ms. Extrastimulus pacing (S_1_-S_4_) and burst pacing (PCL = 50 ms, 2 s) were used to test VT (≥ three consecutive premature ventricular beats) inducibility to all mice. The severity of inducible VT was classified as < 10 beats, between 10 to 30 beats, and > 30 beats^32^.

### Western blotting

Cardiac tissues were sampled from the non-IR and IR zones of the left ventricle at the end of in vivo electrophysiological studies for protein quantification as previously described (n = 6 per group)^33^. See the online supplement for detailed descriptions.

### Langendorff heart preparation and optical mapping studies

Details of the experimental procedure for dual optical mapping of V_m_ and Ca_i_ transients have been described previously^34^. Briefly, the hearts were excised after reperfusion for 10 min and then subjected to Langendorff-perfusion with Rhod-2AM (Ca_i_ indicator), RH237 (V_m_ indicator) and 15 μM blebbistatin (Tocris Bioscience, MN, USA). Epifluorescence was acquired simultaneously using two high-speed cameras (MiCAM Ultima; BrainVision, Tokyo, Japan) at 1 ms/frame. Action potential duration APD_80_ (APD at 80% repolarization) and Ca_i_ alternans were induced and conduction velocity (CV) were studied by a dynamic pacing protocol. VA inducibility was defined as the ability to provoke VT/VF with the dynamic pacing protocol and/or programmed extra stimuli (up to S_4_).

### Cardiomyocyte isolation and whole-cell patch clamp

Cardiomyocytes from the non-IR and IR zones of the left ventricle were isolated using a modified enzymatic digestion protocol (n = 4 per group)^33^. Whole-cell mode of the patch-clamp technique was used to measure *I*_Na_ as described previously^35^. See the online supplement for detailed descriptions.

### Data analysis

APD_80_ and Ca_i_ transient duration at 80% decay (Ca_i_TD_80_) were measured at two PCLs of 200 and 100 ms^24^^.^ The differences between the longest and shortest APD_80_ and Ca_i_TD_80_ were used to represent APD_80_ and Ca_i_TD_80_ dispersion. To estimate CV, we measured the distance and conduction time between the earliest activation point and two epicardial points: one was from the pacing site to the left ventricular apex (CV_IR_), and the other was along an axis parallel to the atrioventricular ring (CV_non-IR_)^36^.

### Statistics

Continuous variables are expressed as mean ± standard deviation and categorical variables are represented by numbers and percentages. One-way analysis of variance (ANOVA) with post hoc least significant difference analysis was performed to calculate statistical significance of differences in continuous variables among four groups. Student’s *t*-test was performed to compare continuous variables between the non-IR and IR zones. Categorical variables were tested using Fisher’s exact test. Differences were considered significant at *P* < 0.05.

### Ethics approval

The present study was approved by Institutional Animal Care and Use Committee of Chang Gung Memorial Hospital (Reference number: 2015092401).

## Supplementary information

Supplementary Legend.

Supplementary Information 1.

Supplementary Information 2.
