# Supplementary material for: Mechanisms of ranolazine pretreatment in preventing ventricular tachyarrhythmias in diabetic db/db mice with acute regional ischemia–reperfusion injury
Source: Sci Rep. 2020 Nov 18;10:20032. doi: 10.1038/s41598-020-77014-0 (PMC7674419; doi:10.1038/s41598-020-77014-0)
Supplement: Supplementary file 1 — Supplementary Legend. [file 41598_2020_77014_MOESM1_ESM.docx]

**Legend for “Western data for ranolazine db mice.xlsx” file:**

The excel file of “Western data for ranolazine db mice.xlsx” shows the quantitative protein expression data normalized to the expression levels of GAPDH, and the histograms representing these normalized densitometric values were shown in Figure 6 and Supplementary Figure S2. CaMKII, calmodulin-dependent protein kinase II; CASQ2, calsequestrin 2; CX43, connexin 43; *db/+* C, control mice not given ranolazine pretreatment; *db/+* R, control mice given ranolazine pretreatment; *db/db* C, diabetic mice not given ranolazine pretreatment; *db/db* R, diabetic mice with ranolazine pretreatment; DHP1α, dihydropyridine receptor 1α; IR, ischemia-reperfusion; NCX, Na^+^-Ca^2+^ exchanger; PLB, phospholamban; PLB-S, pSer16-PLB; PLB-T, pThr17-PLB; SD, standard deviation; SCN5A, voltage-gated Na^+^ channel alpha subunit 5; SERCA, sarcoplasmic reticulum Ca^2+^-ATPase.
