# Supplementary material for: Mechanisms of ranolazine pretreatment in preventing ventricular tachyarrhythmias in diabetic db/db mice with acute regional ischemia–reperfusion injury
Source: Sci Rep. 2020 Nov 18;10:20032. doi: 10.1038/s41598-020-77014-0 (PMC7674419; doi:10.1038/s41598-020-77014-0)
Supplement: Supplementary file 2 — Supplementary Information 1. [file 41598_2020_77014_MOESM2_ESM.pdf]

Mechanisms of Ranolazine Pretreatment in Preventing Ventricular Tachyarrhythmias in Diabetic *db/db* Mice with Acute Regional Ischemia-Reperfusion Injury

Chung-Chuan Chou, MD;[1,6] Hui-Ling Lee, MD;[4] Gwo-Jyh Chang, PhD;[5,6] Hung-Ta Wo, MD;[1] Tzung-Hai Yen, MD;[2,6] Ming-Shien Wen, MD;[1,6] Yen Chu, DSc;[3,6] Hao-Tien Liu, MD;[1] Po-Cheng Chang, MD[1,6]

[1]Division of Cardiology, and [2]Division of Nephrology, Department of Internal Medicine, and [3]Department of Thoracic Surgery, Chang Gung Memorial Hospital, Linkou; [4]Department of Anesthesia, Chang Gung Memorial Hospital, Taipei; [5]Graduate Institute of Clinical Medicine, [6]Chang Gung University College of Medicine, Taoyuan; Taiwan.

Supplementary Methods

This study protocol was approved by the Institutional Animal Care and Use Committee of Chang Gung Memorial Hospital (approval No. 2015092401) and conformed to the Guide for the Care and Use of Laboratory Animals published by the United States National Institutes of Health. Mice were divided into 4 groups: diabetic mice not given ranolazine (*db/db* C, n=22, 12 female, age 23.7 ± 3.6 weeks, body weight 55.0 ± 7.8 g), diabetic mice given ranolazine (*db/db* R, n=21, 11 female, age 23.7 ± 5.5 weeks, body weight 59.6 ± 12.0 g), control mice not given ranolazine (*db/+* C, n=23, 11 female, age 23.2 ± 3.5 weeks, body weight 30.1 ± 3.9 g), and control mice given ranolazine (*db/+* R, n=21, 10 female, age 24.7 ± 2.9 weeks, body weight 31.9 ± 4.2 g). Mice were raised in vivarium under standard conditions at a constant temperature (22 ± 1 ^{o}C), lights on from 8:00 to 20:00, and food and water available ad libitum. Ranolazine (R6152; Sigma-Aldrich, Munich, Germany) was administered orally 305 mg/kg/day (dose comparable with that used clinically in humans of 750 mg twice daily)[1] for 7 days in the ranolazine-given groups.

In-vivo IR model creation and electrophysiological studies

Mice were pre-medicated with xylazine (10 mg/kg intraperitoneally) and zoletil (25 mg/kg intraperitoneally), then intubated and anesthetized with isoflurane using a mouse ventilator (Harvard Apparatus, MA, USA). When the mice were fully anesthetized and unresponsive to physical stimuli, the chests were opened via a median thoracotomy to expose the heart. Regional myocardial ischemia was created using a 7-0 non-absorbable prolene suture line to ligate the left coronary artery at the midway between the atrio-ventricular junction and the apex. Ischemia was confirmed by the appearance of hypokinesis and pallor distal to the occlusion. After 15 minutes of ischemia, the ligature was removed, and reperfusion was visually confirmed (Figure 5A).

In vivo electrophysiological study was performed using the same method previously described.[2] Platinum electrodes were inserted into the limbs and three-lead pseudo-electrocardiogram was obtained with Axon Digidata (Molecular Devices, CA, USA). A custom-made bipolar electrode was placed on the ventricular epicardium for ventricular pacing at twice the threshold. Extrastimulus pacing (S_1-S_4) and burst pacing (pacing cycle length (PCL) = 50 ms, 2 sec) were used to test VT (≥ three consecutive premature ventricular beats) inducibility to all mice. We first measured effective refractory period by giving a premature stimulus after 8 beats of S_1S_1 pacing at a PCL of 200 ms. The extrastimulus pacing represents a fixed S_1S_1 PCL of 200 ms for 8 beats, followed by extrastimuli from 80 ms in 10 ms decrements. The S_1-S_2 and S_2-S_3 intervals were reduced with either the S_1-S_2 being long and S_2-S_3 being short or vice versa, or be decreased simultaneously. The S_3-S_4 interval was shortened until loss of ventricular capture or induction of VT. The severity of inducible VT was classified as < 10 beats, between 10 to 30 sec, and > 30 beats.

Western blotting

Cardiac tissues were sampled from the non-IR and IR zones of the mapped anterior aspect of left ventricle at the end of in vivo electrophysiological studies for protein quantification as previously described (n = 6 per group).[3] Briefly, 50 μg of protein was loaded to each well in the SDS-PAGE gel for electrophoresis. The sample was then transferred onto a polyvinylidene difluoride membrane (PVDF, Immobilon-P, EMD millipore, Temecula, CA, USA). Primary antibodies against SERCA2a (Thermo, USA), CaMKIIδ (Cell Signaling, USA), $pThr^{287}$-CaMKIIδ (CaMKIIδ-p, Thermo, USA), PLB (phospholamban, Cell Signaling, USA), $pSer^{16}$-phospholamban (PLB-S, Gene Tex, USA), $pThr^{17}$-PLN (PLB-T, Badrilla, UK), dihydropyridine receptor (DHP1α, Abcam, Cambridge, UK), Na^{+}-Ca^{2+} exchanger (NCX, Abcam, Cambridge, UK), calsequestrin 2 (CASQ2) (Gene Tex, USA), Nav1.5 (SCN5A) (Alomone, USA), connexin43 (Cx43, Gene Tex, USA), and GAPDH (Santa Cruz, USA) were used to detect the proteins of interest. Secondary antibodies, such as goat anti-mouse IgG-HRP (Leinco Technologies, USA), goat anti-rabbit IgG-HRP (Leinco Technologies, USA) and donkey anti-goat IgG-HRP antibody (Santa Cruz, USA), were used in conjunction with primary antibodies. Signals were obtained through enhanced chemiluminescence (Pierce ECL Western Blotting Substrate, Thermo, USA) and blots were quantified through scanning densitometry. The levels of protein expression were normalized to that of GAPDH. We performed several scans with different exposure, and Figure S3-3 shows an example of the PLB gels with different exposure.

Langendorff heart preparation and optical mapping studies

Details of the experimental procedure for dual optical mapping of membrane voltage (V_m) and Ca_i transients have been previously described.[4] In brief, the hearts were excised after

reperfusion for 10 min, Langendorff-perfused and loaded with Rhod-2AM (Ca_i indicator) and RH237 (V_m indicator). Motion artifacts were suppressed using 15 μM blebbistatin (Tocris Bioscience, MN, USA). The hearts were illuminated using a solid-state, frequency-doubled laser light source (Millennia, Spectra-Physics Inc., CA, USA) with a wavelength of 532 nm. Epifluorescence was acquired simultaneously through two high-speed cameras (MiCAM Ultima, BrainVision, Tokyo, Japan) at 1 ms/frame through a 580 ± 20-nm bandpass filter and a 715-nm long-pass filter for Ca_i and V_m images, respectively. Digital images (100 × 100 pixels) were gathered from a mapped field of 14×14 mm^2, resulting in a spatial resolution of 140×140 μm^2 per pixel. A bipolar lead was used to pace the lateral wall of the left ventricle at a double threshold. The effective refractory period (ERP) was measured by giving a premature stimulus after 8 beats at a 200-ms PCL. Action potential duration $(APD)_{80}$ (APD at 80% repolarization) and Ca_i alternans were induced and conduction velocity (CV) were studied by a dynamic pacing protocol.[4] VA inducibility was defined as the ability to provoke VT/VF with the dynamic pacing protocol and/or programmed extra stimuli (up to S_4).

Cardiomyocyte isolation and whole-cell patch clamp

Cardiomyocytes from left ventricular non-IR and IR zones were isolated using a modified enzymatic digestion protocol.[5] Following the IR injury, the mouse hearts were harvested and Langendorff-perfused for 10 minutes with I_{Na} bath solution. The composition of the I_{Na} bath solution consisted of (in mmol/L) NaCl, 137; KCl, 5.4; $MgSO_4$, 1.22; NaH_2PO_4, 1.2; HEPES, 6; D-glucose, 22; and $CaCl_2$, 1.8 (pH 7.4 with NaOH). The hearts were then perfused with Ca^{2+}-free I_{Na} bath solution for another 10 minutes. The hearts were then digested with the same I_{Na} bath solution containing 150 U/mL type II collagenase (Worthington, Lakewood, NJ, USA) for 30 minutes. After the enzymatic digestion, the hearts were removed from the perfusion system. Tissues from the left ventricular IR and non-IR zones were cut into small

pieces and dissected mechanically to obtain isolated cardiomyocytes. All enzymatic isolation procedures were performed at 37°C. During the procedure, the hearts were maintained at a perfusion pressure at 80 cmH_2O.

Whole-cell mode of the patch-clamp technique was used to measure late I_{Na} as described previously.[6] All experiments were conducted at room temperature. An Axopatch 200B amplifier was used to generate pulse protocols. A software pCLAMP 8 (Molecular Device/Axon, Sunnyvale, CA, USA) was used to record data. Borosilicate glass capillaries (WPI Inc., Sarasota, FL, USA) were used to make pipette electrodes with a resistance of 3–5 MΩ in the bath solution. The pipette solution contained (in mmol/L): CsCl, 130; TEA-Cl, 15; HEPES, 10; D-glucose, 5 and EGTA, 5 (pH 7.4 with CsOH). Current traces were analyzed using Clampfit- 8 (Molecular Devices/Axon). Whole-cell configuration was performed in Ca^{2+}-free I_{Na} bath solution (with additional 2 mmol/L CsCl), and capacitance currents were monitored with a repetitive 5-mV step pulse for at least 5 minutes. Whole cell currents were elicited by a 300-ms depolarizing pulse from a holding potential of -80 to 30 mV at a frequency of 0.2 Hz in the ventricular myocytes. The amplitude of late I_{Na} was measured at 200 ms after a depolarizing pulse to eliminate the effect of transient sodium current. Current density was calculated by dividing the amplitude by the cell capacitance. All chemicals were purchased from Sigma unless otherwise stated (Sigma, St. Louis, MO, USA).

Data analysis

APD_{80} and Ca_i transient duration (Ca_iTD_{80}) were measured at PCLs of 200 and 100 ms using the same method as previously described.[2] We used monoexponential fittings to compute the time constant (τ) of the decay portion of the Ca_i transient between 70% of the transient peak and the diastolic baseline. The thresholds of APD_{80} and Ca_i alternans were defined as the

longest PCL required to produce a 4-ms difference in APD_{80} and a 10% difference in Ca_i amplitude between consecutive beats, respectively. The phase was considered positive for a short-long APD and a small-large Ca_i amplitude sequence (color coded in red) and was negative for a long-short APD and a large-small Ca_i amplitude sequence (color coded in green). Spatially discordant alternans (SDA) was evidenced by the presence of both red and green regions separated by a nodal line. To estimate CV, we measured the distance and conduction time between the earliest activation point and two epicardial points: one was from the pacing site to the LV apex (CV_{IR}), and the other was along an axis parallel to the atrioventricular ring ($CV_{non\text{-}IR}$) (Figure 4B, right subpanels).[7]

References

1. Tocchetti CG, Carpi A, Coppola C, Quintavalle C, Rea D, Campesan M, Arcari A, Piscopo G, Cipresso C, Monti MG. Ranolazine protects from doxorubicin- induced oxidative stress and cardiac dysfunction. European journal of heart failure 2014;16:358-366.

2. Chou CC, Ho CT, Lee HL, Chu Y, Yen TH, Wen MS, Lin SF, Lee CH, Chang PC. Roles of impaired intracellular calcium cycling in arrhythmogenicity of diabetic mouse model. Pacing and Clinical Electrophysiology 2017;40:1087-1095.

3. Chang PC, Huang YC, Lee HL, Chang GJ, Chu Y, Wen MS, Chou CC. Inhomogeneous Down- regulation of INa underlies piceatannol pro- arrhythmic Mechanism in regional ischemia- reperfusion. Pacing and Clinical Electrophysiology September 2018 2018;41:1116-1122.

4. Chou CC, Chang PC, Wei YC, Lee KY. Optical mapping approaches on muscleblind-like compound knockout mice for understanding mechanistic insights into ventricular arrhythmias in myotonic dystrophy. Journal of the American Heart Association 2017;6:e005191.

5. Chang PC, Turker I, Lopshire JC, Masroor S, Nguyen BL, Tao W, Rubart M, Chen PS, Chen Z, Ai T. Heterogeneous upregulation of apamin- sensitive potassium currents in failing human ventricles. Journal of the American Heart Association 2013;2:e004713.

6. Chang G-J, Chang C-J, Chen W-J, Yeh Y-H, Lee H-Y. Electrophysiological and mechanical effects of caffeic acid phenethyl ester, a novel cardioprotective agent with antiarrhythmic activity, in guinea-pig heart. European journal of pharmacology 2013;702:194-207.

7. Chou C-C, Chang P-C, Wen M-S, Lee H-L, Wo H-T, Yeh S-J, Wu D. Piceatannol

facilitates conduction block and ventricular fibrillation induction in ischemia-reperfused rabbit hearts with pacing-induced heart failure. International journal of cardiology 2014;171:250-258.

Supplementary Figure S1

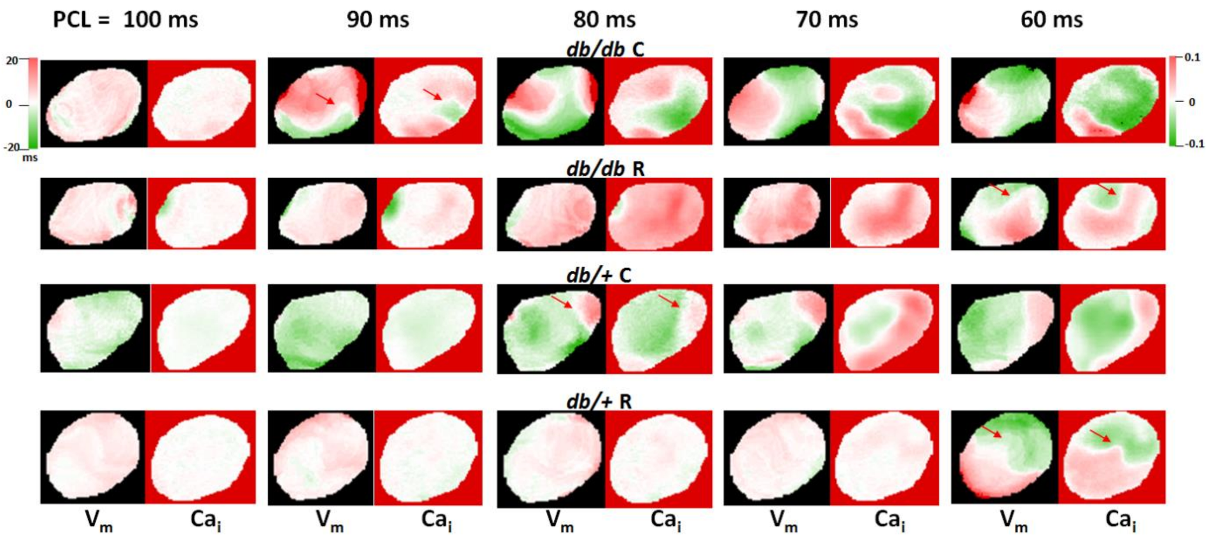

Supplementary Figure S1 Representative examples of membrane voltage (V_m) and intracellular Ca^{2+} (Ca_i) alternans maps in the four study groups. Spatially discordant alternans (SDA) was induced by shortening pacing cycle length (PCL). Ranolazine increased the pacing threshold of SDA induction. Red arrows indicate the nodal lines.

Supplementary Figure S2

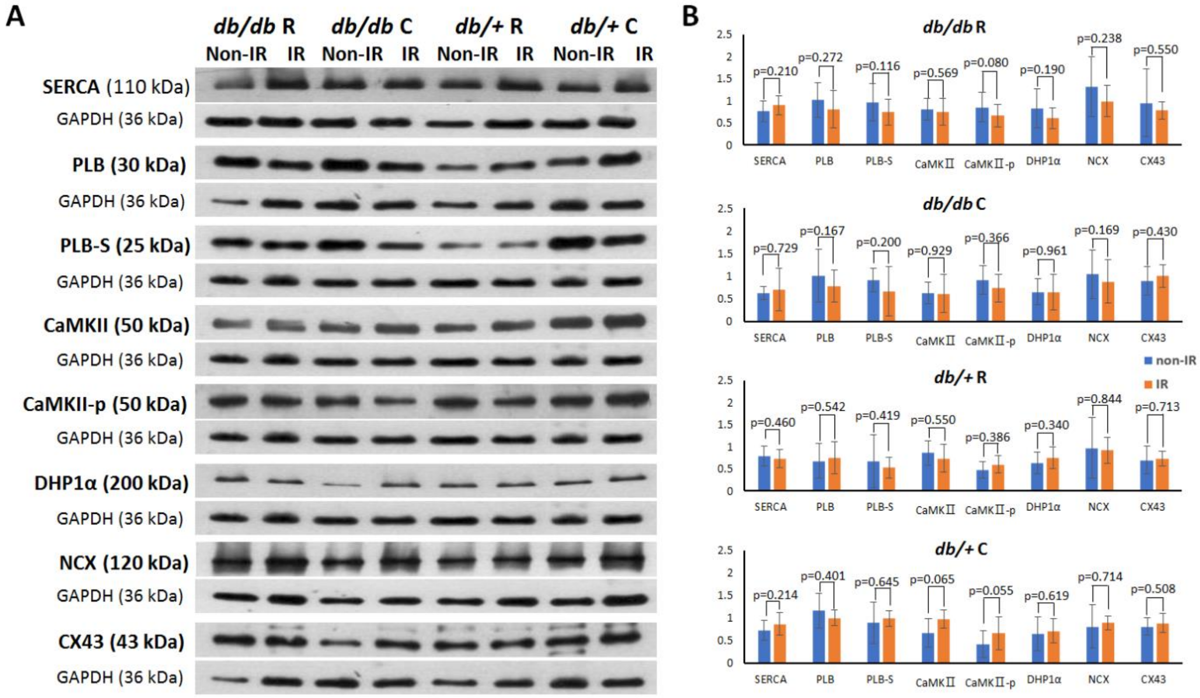

Supplementary Figure S2 Representative examples of Western blotting results. Panels show representative bands (A) and histograms represent densitometric values normalized to the corresponding GAPDH (B). Values are means, with their standard errors represented by vertical bars.

Supplementary Figure S3-1

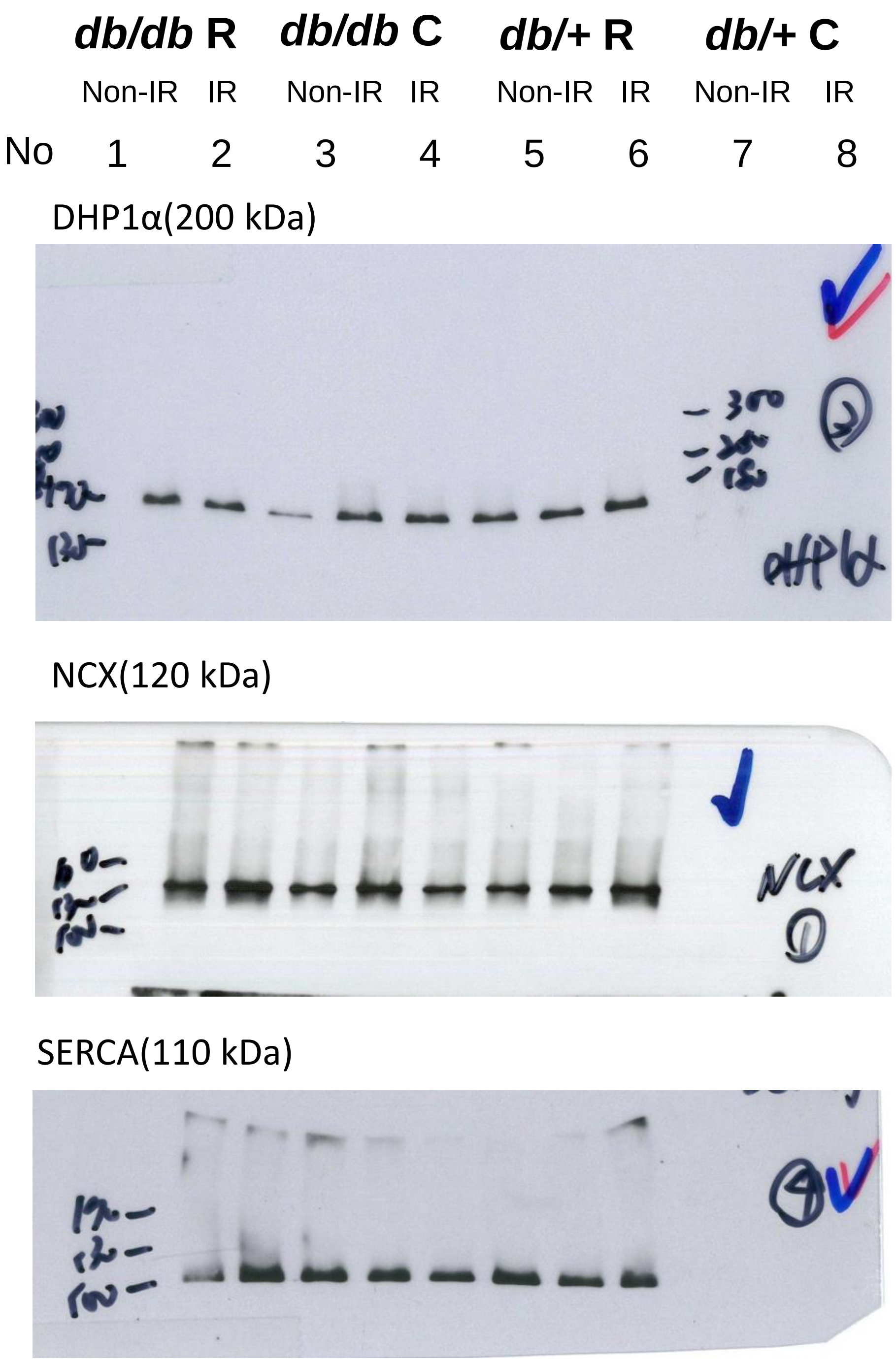

Supplementary Figure S3-2

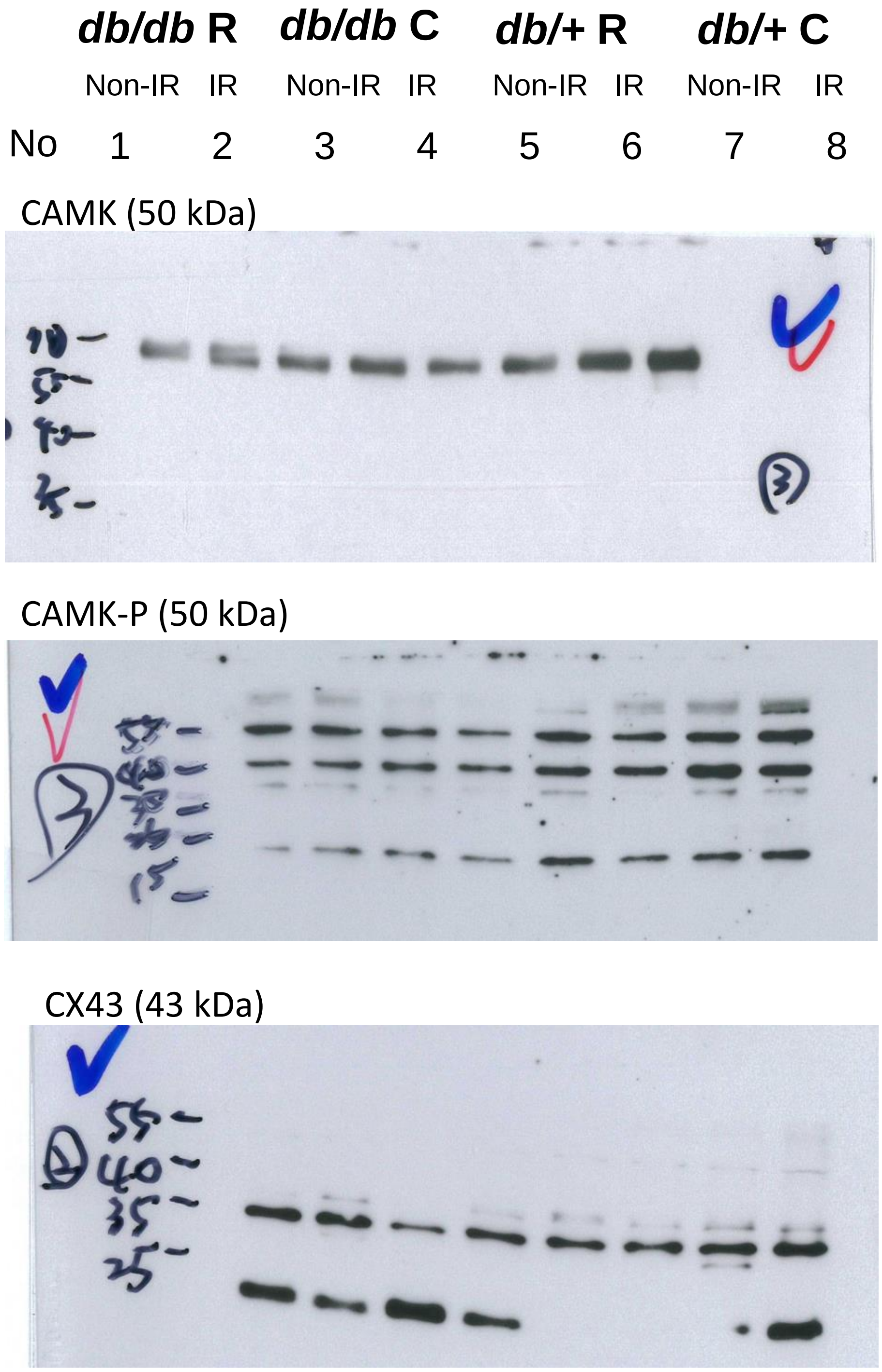

Supplementary Figure S3-3

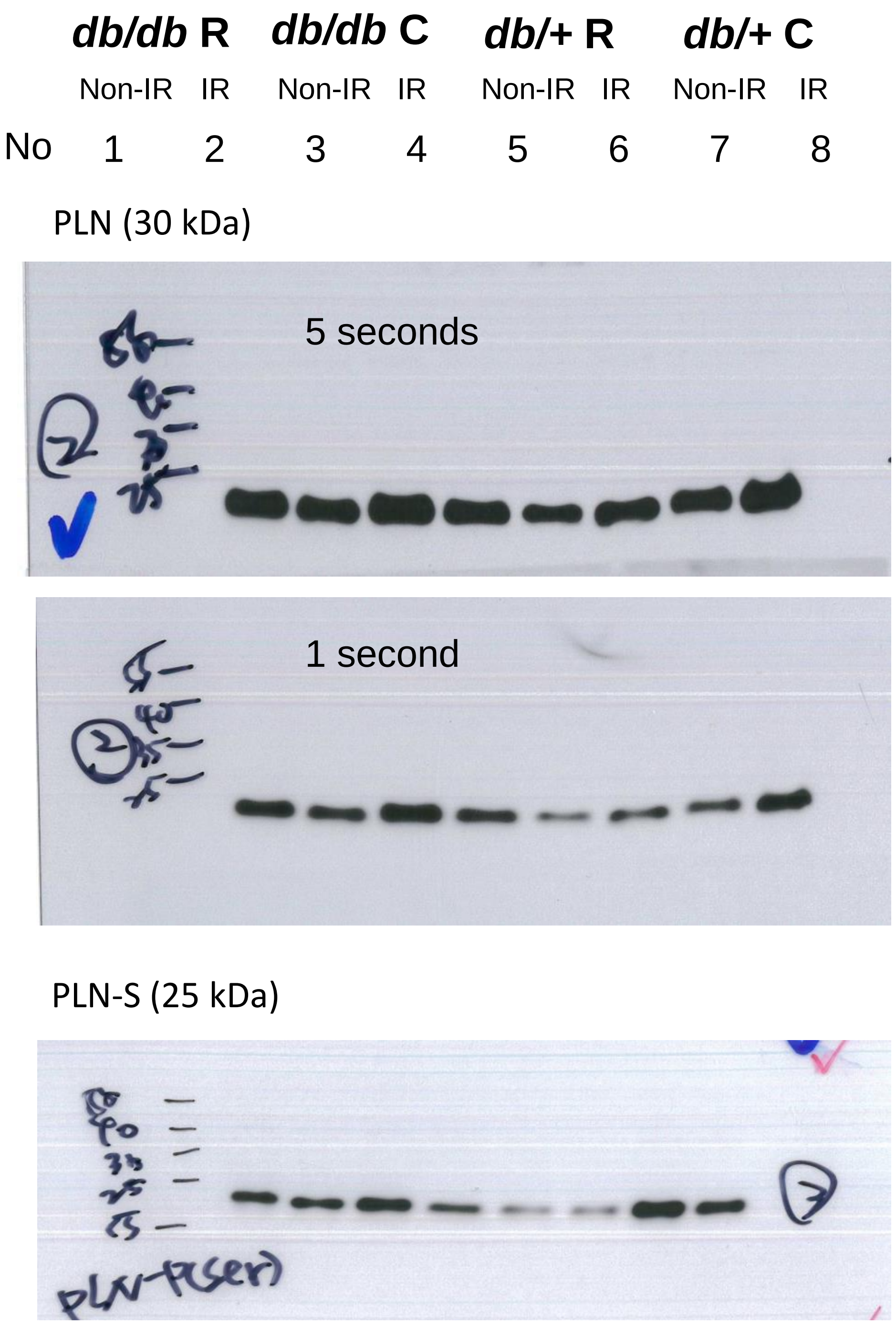

Supplementary Figure S3-4

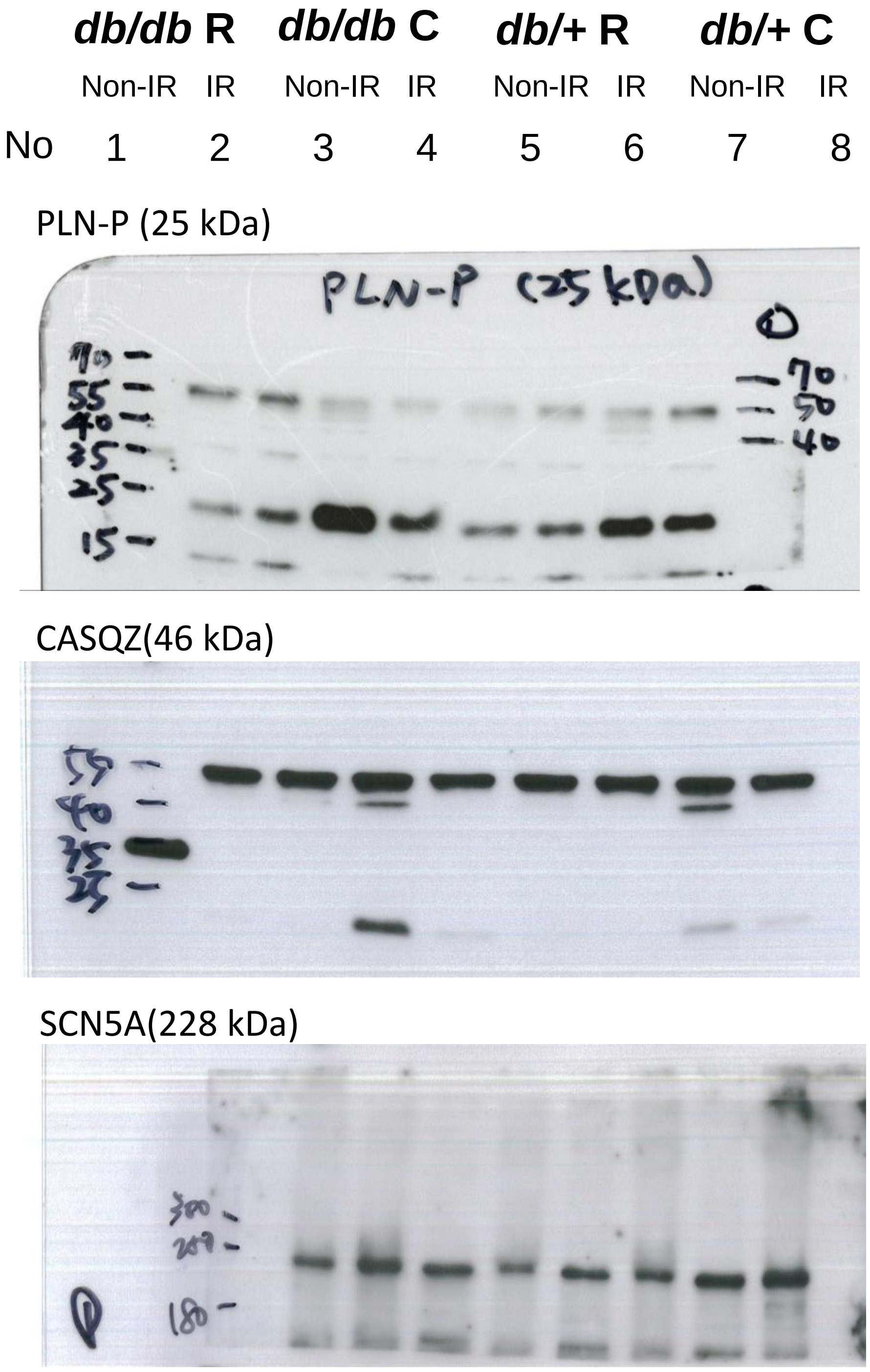

Supplementary Figure S3-5

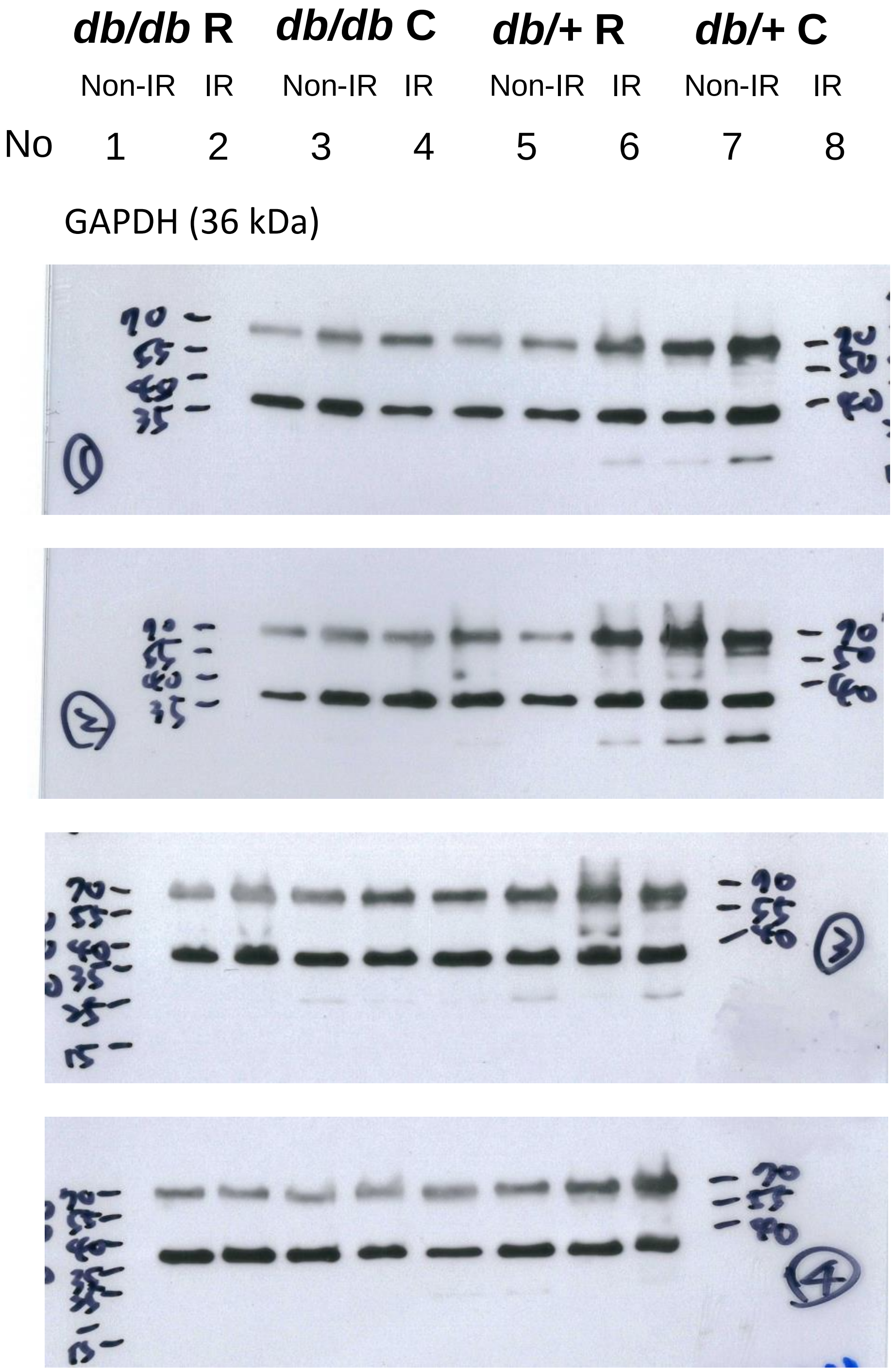

Supplementary Figure S3. Representative examples of the original gel of the Western blotting. Grouping and the corresponding serial numbers were displayed in each cropped gel. All the illustrations were acquired from the original gels without editing. Figure S3-3 shows examples of PLB bands with 5-sec (adequate) exposure and 1-sec exposure.
